# Structure, phylogeny, allelic haplotypes and expression of sucrose transporter gene families in *Saccharum*

**DOI:** 10.1186/s12864-016-2419-6

**Published:** 2016-02-01

**Authors:** Qing Zhang, Weichang Hu, Fan Zhu, Liming Wang, Qingyi Yu, Ray Ming, Jisen Zhang

**Affiliations:** Center for Genomics and Biotechnology, Haixia Institute of Science and Technology (HIST), Fujian Agriculture and Forestry University, Fuzhou, 350002 China; Department of Plant Biology, University of Illinois at Urbana-Champaign, Urbana, IL 61801 USA; TexasA&M AgriLife Research, Department of Plant Pathology and Microbiology, Texas A&M University System, Dallas, TX 75252 USA; College of Life Sciences, Fujian Normal University, Fuzhou, 350117 China

**Keywords:** *Saccharum* species, Sucrose transporter, Gene family, Allelic haplotype, Polyploidy

## Abstract

**Background:**

Sugarcane is an economically important crop contributing to about 80 % of the world sugar production. Increasing efforts in molecular biological studies have been performed for improving the sugar yield and other relevant important agronomic traits. However, due to sugarcane’s complicated genomes, it is still challenging to study the genetic basis of traits, such as sucrose accumulation. Sucrose transporters (SUTs) are critical for both phloem loading in source tissue and sucrose uptaking in sink tissue, and are considered to be the control points for regulating sucrose storage. However, no genomic study for sugarcane sucrose transporter (SsSUT) families has been reported up to date.

**Results:**

By using comparative genomics and bacterial artificial chromosomes (BACs), six SUT genes were identified and characterized in *S. spontaenum*. Phylogenetic analyses revealed that the two pairs *SsSUTs* (*SsSUT1*/*SsSUT3* and *SsSUT5*/*SsSUT6*) could be clustered together into two separate monocot specific SUT groups, while *SsSUT2* and *SsSUT4* were separated into the other two groups, with members from both dicot and monocot species. Gene structure comparison demonstrated that the number and position of exons/introns in *SUTs* were highly conserved among the close orthologs; in contrast, there were variations among the paralogous *SUTs* in *Sacchuarm*. Though with the high polyploidy level, gene allelic haplotype comparative analysis showed that the examined four *SsSUT* member*s* exhibited conservations of gene structures and amino acid sequences among the allelic haplotypes accompanied by variations of intron sizes. Gene expression analyses were performed for tissues from seedlings under drought stress and mature plants of three *Saccharum* species (*S.officinarnum*, *S.spotaneum* and *S.robustum*). Both *SUT1* and *SUT4* expressed abundantly at different conditions. *SUT2* had similar expression level in all of the examined tissues, but *SUT3* was undetectable. Both of *SUT5* and *SUT6* had lower expression level than other gene member, and expressed stronger in source leaves and are likely to play roles in phloem loading. In the seeding plant leave under water stress, four genes *SUT1*, *SUT2*, *SUT4* and *SUT5* were detectable. In these detectable genes, *SUT1* and *SUT4* were down regulated, while, *SUT2* and *SUT5* were up regulated.

**Conclusions:**

In this study, we presented the first comprehensive genomic study for a whole gene family, the SUT family, in *Saccharum*. We speculated that there were six *SUT* members in the *S. spotaneum* genome. Out of the six members, *SsSUTs*, *SsSUT5* and *SsSUT6* were recent duplication genes accompanied by rapid evolution, while, *SsSUT2* and *SsSUT4* were the ancient members in the families. Despite the high polypoidy genome, functional redundancy may not exist among the SUTs allelic haplotypes supported by the evidence of strong purifying selection of the gene allele. *SUT3* could be a low active member in the family because it is undetectable in our study, but it might not be a pseudogene because it harbored integrated gene structure. *SUT1* and *SUT4* were the main members for the sucrose transporter, while, these *SUTs* had sub-functional divergence in response to sucrose accumulation and plant development in *Saccharum*.

**Electronic supplementary material:**

The online version of this article (doi:10.1186/s12864-016-2419-6) contains supplementary material, which is available to authorized users.

## Background

Sucrose transporters (SUTs) are important for both phloem loading in source tissue and sucrose uptake into some sink cells [1]. SUTs are considered to be the control points for sucrose storage in plant because they can carry sucrose across cell membranes and play an important role in loading sucrose into phloem systems in a series of steps [1]. Plant SUTs belong to the glycoside-pentoside-hexuronide (GPH) cation symporter family (TC2.A.2) that is part of the major facilitator super-family (MFS) [2]. Transporters in the GPH family have the primary characteristics of MFS proteins that contain 12 transmembrane spanning domains with N- and C-termini in the cytoplasm [3]. Since the first plant sucrose transporter SoSUT1 was functionally identified using an elegant yeast complementation strategy from spinach (*Spinacea Oleracea*) [4], SUTs have been demonstrated to effect on the multiple aspects of plant development such as biomass partitioning, pollen germination, restraining plant growth, fruit size reduction and ethylene biosynthesis [5–9].

Comprehensive understanding of the molecular structure and evolution of a gene family in plant species is the first step towards understanding their physiological roles and metabolic mechanism involved in different growth phases. Recent studies have revealed that *SUT* was a small gene family that consisted of at least four *SUT* genes in the most plant species. The SUT family members have been identified from a number of plant species such as *Arabidopsis* [10], rice (*Oryza sativa*) [11], wheat (*Triticum aestivum*) [12], populus [8], sorghum (*Sorghum bicolor*) [13] and pineapple (*Ananas comosus*) (Unpublished, Zhang and Ming) as well. Phylogenetic analysis of *SUT* family suggested that the plant *SUT* could be divided into five subgroups including two monocot specific subgroups, one dicot specific and two monocot-dicot subgroups. *SUT* family members in many plant species are divergent in function and deferentially expressed in different tissues types or at different plant developmental stages. For instance, in rice, *OsSUT1* expression has been detected in germinated seeds, leaf blades, leaf sheaths and panicles [14–16]; the expressions of both *OsSUT3* and *OsSUT5* are dramatically lower in embryos than those of *OsSUT1*, *OsSUT2,* and *OsSUT4* [17]; *OsSUT4* has been detected in most tissues such as roots, leaves, and panicles [11], and could play a role in sucrose loading into the sheath phloem of the upper leaves during the post-heading period for sucrose transport to developing grains [18]. In *Arabidopsis* [10], *AtSUC2* has been detected in phloem and companion cells in source leaves [19, 20] and functions in loading sucrose into the phloem sieve elements (SEs), correspond with the result identified by tissue-specific complementation of different promoters [7, 21] and by ^14^C labeling studies [22]; *AtSUC3* and *AtSUC4* are expressed in minor veins of source leaves of mature plants [23–25].

Sugarcane (*Saccharum spp.)* is one of the world’s most produced crops (FAOSTAT, 2015), and contributes to about 80 % of the world sugar and about 40 % of ethanol production worldwide. Modern sugarcane cultivar has one of the most complex genome among all the crops; by being both aneupoid and autopolypoid with an extreme ploidy level that can range from octoploidy (x = 8) to dodecaploidy (x = 12). Approximately 80 % of cultivars’ chromosomes are derived from *S. officinarum* and 10–20 % is derived from *S.spontaneum* with the remained from interspecific recombination [26–28]. Sugarcane is not only an economically important crop species, but also serves as an important model crop for studying sucrose transporters because its remarkable ability to accumulate vast amounts of sucrose in its stems that can reach close to 700 mM or in excess of 50 % of the dry weight (DW) (DW) [29]. But to date, limited works in characterizing these *SUT* genes have been reported, except for *SUT1* (transcripts accession:AY780256.1, GU812864.1 and BU925792). In an earlier study based on sugarcane Expressed Sequence Tag (EST) database survey, *SUT1* was revealed to be more abundant in the mature internodes than the other tissues [30]. This result was confirmed by a study for Hawaiian sugarcane cultivar, in which the *SUT1* transcript levels increased during maturation and sucrose storage, whereas, *SUT1* expression was observed to be not affected by sugarcane yellow leaf virus (SCYLV) infection in sugarcane [31]. Biochemical analysis of sugarcane *SUT1* suggested that SUT1 was highly selective for sucrose, but had a relatively low affinity for sucrose, inhibited by sucralose and played key role in sucrose loading from the vascular tissues into the storage sites in parenchyma cells of sugarcane stems [32, 33]. Besides *SUT1*, the gene family of *SUTs* is not understood in sugarcane due to the formidable challenge caused by its high degree of polyploidy and heterozygosity genome.

In this study, to gain comprehensive understandings of the molecular and evolutionary characterization as well as the possible functions of *SUT* family in sugarcane, based on combination of comparative genomics strategies and high genome coverage of bacterial artificial chromosomes (BACs) libraries resources, we identified and characterized *SUT* gene families in *Saccharum* species and investigated their transcriptional expression patterns. The analysis in this study mainly focused on: (1) identifying the gene members and allele haplotypes of the *SUT* gene family in sugarcane; (2) analyzing evolutionary relationship, exon/intron organization of the *SUT* gene family; and (3) characterizing the expression patterns of the SUT gene family in three progenitor *Saccharum* species.

## Methods

### Plant materials

Three varieties of *Saccharum* species were used in the study: LA-Purple (*S. officinarum*, 2n = 8x = 80), Molokai6081 (*S. robustum*, 2n = 8x = 80) and SES208 (*S. spontaneum*, 2n = 8x = 64) [34]. Plants were grown in plastic pots under greenhouse conditions and standard growing practices. Tissue samples were obtained from 10-month old plants (as replicates) for leaf roll, leaf, top immature internode (i.e. internode number 3), premature internode (i.e. internode number 9 for ‘LA Purple’ and Molokai6081 due to short internode, and internode number 6 for SES208 due to long internode) and mature internode (i.e. internode number 15 for ‘LA Purple’ and Molokai6081, and internode number 9 for SES208 due to long internode – most SES208 plants have about 12 internodes). The internodes were numbered from top to bottom according to the method of Moore [35]. Stem and leaf tissues from seedlings of the three species were collected at 35 days after planting. For drought stress treatment, the 35 day-old seedlings were treated with PEG6000 (30 %) for 48 h, and stem and leaf tissues were collected. The tissues were immediately frozen using liquid nitrogen and stored at −80 °C prior to RNA isolation.

### BAC libraries

The haploid of *S. Spontaneum* SES208, Ap85-441 (2n = 4x = 32), was used to construct the BAC library. Nuclei were isolated from the young leaf tissues of AP85-441 following the method described by Ming *et al*. [36]. The high molecular weight DNA embedded in agarose was partially digested using *HindIII*. Fractions at approximately 100 kb were recovered and cloned into pSMART BAC vector (Lucigen, LA). 38,400 clones from AP85-441 BAC libraries were picked and stored in 100 384-well plates with freezing medium. BAC clones were grided onto Performa II Nylon Filters (Genetix) using Q-Pix 2 (Genetix).

### Database search for the SUT gene family and phylogenetic analyses

The sequence data used in this study were collected using the keyword “sucrose transporter” and a query search in the GenBank using the known SUT gene sequences from sorghum [13], rice [11] and *Arabidopsis* [24]. Matches achieved similarity scores of 50.0 and probability scores >50.0 and e-value <10^−4^ were collected.

The amino acid sequences of sucrose transporter gene family members in 6 monocotyledons (*Zea mays*, *Sorghum bicolor*, *Oryza sativa*, *Brachypodium distachyon*, *Setaria italica* and *Saccharum spontaneum*) and 5 dicotyledons (*Arabidopsis thaliana*, *Citrus sinensis*, *Glycine max*, *Solanum tuberosum* and Vitis vinifera,) identified by searching public databases available at various resources. The phylogenetic trees were constructed with the MEGA5.2.1 program with ClustalW alignment using default parameter.

### Identification and sequencing of SUT families from BAC library

BAC library screening was carried out as described by Yu *et al.*[37]. The BAC clones representing different haplotypes were selected. The insert size of BAC clones was estimated by comparing with standard size markers using CHEF gel electrophoresis. The BAC DNAs were isolated using PhasePrep^TM^ TMBAC DNA kit (Sigma-Aldrich, NA0100-1KT) and the sequencing libraries were prepared individually with unique barcode for each clone. The sequencing libraries were then pooled and sequenced with 150 bp, pair-end reads on Illumina Hiseq2500 at Center for Genomics and Biotechnology in Fujian Agriculture and Forestry University. The raw reads were then assembled using SPAdes Genome Assember v. 3.1.1 (http://bioinf.spbau.ru/en/spades).

### Genomic sequence annotation and functional prediction

The genomic sequences of SUT genes were annotated by DNA subway (http://dnasubway.iplantcollaborative.org/), and the corresponding CDS sequences were translated into protein by the EXPASy-translate tool (http://web.expasy.org/translate/). The exon-intron structures were graphed using online tool GSDS (http://gsds.cbi.pku.edu.cn/). The putative conserved domains of sucrose transporter protein were detected by using BLASTp (http://blast.ncbi.nlm.nih.gov/Blast.cgi) and InterPro (http://www.ebi.ac.uk/interpro/scan.html). The protein transmembrane helices domain was predicted using TMPRED (http://www.ch.embnet.org/software/TMPRED_form.html). The isoelectric point and relative molecular mass of the protein were predicted using ExPASy (http://web.expasy.org/compute_pi/).

### Analysis of sucrose transporter gene co-expression profiling

The sorghum gene models were used as reference to align the *Saccharum* RNA-seq database by using NOVOALIGN (http://www.novocraft.com/) with default parameter. The normalization and statistical evaluation of differential gene expression has been performed using EDGE-R with a *p*-value cut-off of 0.05 and using the Benjamini-Hochberg (1995)[38] method for multiple testing corrections. The raw data was normalized according to the default procedure of the differential expression analysis package used. The dispersion was estimated using the pooled setting. The expression values were log-transformed, and cluster analyses were performed using a software cluster with Euclidean distances and the hierarchical cluster method of “complete linkage clustering”. The clustering tree was constructed and viewed in JAVA Treeview.

### Experimental validation of expression levels of SUT gene by qRT-PCR

The expression levels of six SUT genes in three tissues (internode 9, 15 and leaf roll in LA-Purple, internode 8,13 and leaf roll in Molokai6081, and internode 6, 9 and leaf roll in SES208) of three Saccharum species were validated by qRT-PCR. Gene-specific primer pairs were designed by using Integrated DNA Technologies (IDT) (http://www.idtdna.com/Primerquest/Home/Index). After treated with DNase I (Tiangen, China), two microgram of RNA was used in reverse transcription with the SuperScript VILO cDNA Synthesis Kit (Invitrogen) according to the manufacturer’s guidelines. The real-time qPCR was performed by using Multicolor Real-Time PCR Detection System (Bio-Rad) with conditions for all reactions were 95 °C for 30s, 40 cycles of 95 °C for 5 s, followed by 60 °C for 30s, and 95 °C for 10s. Melting curve analysis were performed to confirm the PCR specificity. The glyceraldehyde-3-phosphate dehydrogenase gene (*GAPDH*) and Eukaryotic elongation factor 1a (*eEF-1a*) were selected as internal standard for normalization [39], and three replicates were completed for each sample. The relative expression level for each SUT gene in different tissues of three Saccharum species were calculated by using the 2-ΔΔCt method. The correlation coefficient was calculated between the transcript accumulation levels obtained by RNAseq and qRT-PCR using Excel.

## Results

### Identification of six SUT genes in Sugarcane

20 positive BAC clones from AP85-441 were identified using 6 probes (Additional file 1) designed from six well-annotated *SUTs* genomic regions in *Sorghum bicolor* (Table 1). To determine haplotypes, the PCR fragments of the six *SUTs* were cloned by using these probe primers and sequenced, which confirmed 14 of these 20 BAC clones contains different paralogous and homologous haplotypes. The six *S.spontaneum SUTs* were referred to *SsSUT1*-*SsSUT6* according to sequence similarity with sorghum *SUTs* [13]. In the 14 *SUT* sequences, both *SsSUT1* and *SsSUT5*, both *SsSUT3* and *SsSUT6* and remaining two *SUTs* have 2, 4 and 1 allelic haplotypes, respectively. The allelic haplotypes of each *SUTs* were indicated additional“-h1” to “-h4” to the gene name end. Using the gene model sequences of the annotated *SUT* genes as queries, both the in-house EST and Genbank database were extensively searched. The results showed that all the *SsSUTs* had the corresponding ESTs in the Genbank database except *SsSUT3* (Additional file 2).

**Table 1:** Information of the putative SUT genes in sorghum

Gene name	Gene ID	Location of the gene
*SbSUT1*	*Sb*01g045720	NC_012870.1|:68807992-68813945 chromosome 1
*SbSUT2*	*Sb*04g038030	NC_012873.1|:67544380-67548965 chromosome 4
*SbSUT3*	*Sb*01g022430	NC_012870.1|:c28297080-28293801 chromosome 1
*SbSUT4*	*Sb*08g023310	NC_012877.1|:c55444565-55438275 chromosome 8
*SbSUT5*	*Sb*04g023860	NC_012873.1|:c53548702-53545522 chromosome 4
*SbSUT6*	*Sb*07g028120	NC_012876.1|:c63108611-63106213 chromosome 7

The six *SsSUTs* containing complete ORFs (open reading frames) with the predicted molecular weights ranged from 51.84 to 63.41 kDa in sugarcane (Table 2). Comparing with the *SUT* family from sorghum, *SsSUT5* showed a lower molecular weight, and the remaining gene pairs between these two species were consistent. *SsSUT5* and *SsSUT6* shared a higher similarity of protein sequences (82 %) in contrast to the other 14 pairwise sequences between the remaining four genes in the SUT families (39–69 %) (Table 3). The analyses of the deducted protein sequences of the *SUT* genes in sugarcane revealed that all these gene families had highly conserved MFS domains and contained 12 membrane-spanning helices (Additional file 3). A conserved histidine residue was presented in the first loop domain corresponding to His-65 [40] and amino acids which corresponded to the G-X-X-X-D/E-R/K-X-G-[X]-R/K-R/K motif reside in the second and eighth loop domains [41, 42]. Additionally, *SsSUT4* contained a LXXLL motif in the N-terminal domain, indicating that it might be targeted to the tonoplast [13, 43].

**Table 2:** Comparison of the characterization of the SUTs between sugarcane and sorghum

Sorghum						Sugarcane						
Gene name	Amino acids size	Molecular weights (kDa)	Domains	Isoelectric point (pI)	Transmembrane helices	Gene name	Amino acids size	Molecular weights (kDa)	Domains	Isoelectric point (pI)	Transmembrane helices	Identity
*SbSUT1*	519	54.99	MFS domain	8.86	12	*SsSUT1*	521	55.08	MFS domain	8.79	12	96 %
*SbSUT2*	594	63.33	MFS domain	6.00	12	*SsSUT2*	598	63.41	MFS domain	5.94	12	96 %
*SbSUT3*	507	53.20	MFS domain	6.58	12	*SsSUT3*	508	53.47	MFS domain	7.46	12	96 %
*SbSUT4*	501	53.44	MFS domain	8.60	12	*SsSUT4*	501	53.44	MFS domain	8.60	12	98 %
*SbSUT5*	534	56.38	MFS domain	8.72	12	*SsSUT5*	495	51.84	MFS domain	7.97	12	83 %
*SbSUT6*	536	56.36	MFS domain	8.45	12	*SsSUT6*	554	58.86	MFS domain	8.46	12	88 %

**Table 3:** Amino acid sequences pairwise comparisons (% similarity) between SUT gene members in sugarcane

	*SsSUT1*	*SsSUT2*	*SsSUT3*	*SsSUT4*	*SsSUT5*	*SsSUT6*
*SsSUT2*	55 %	-	-	-	-	-
*SsSUT3*	69 %	53 %	-	-	-	-
*SsSUT4*	47 %	43 %	45 %	-	-	-
*SsSUT5*	50 %	43 %	49 %	39 %	-	-
*SsSUT6*	53 %	47 %	53 %	41 %	82 %	-

### Allelic haplotype analysis of *SsSUTs*

**Fig. 1:**
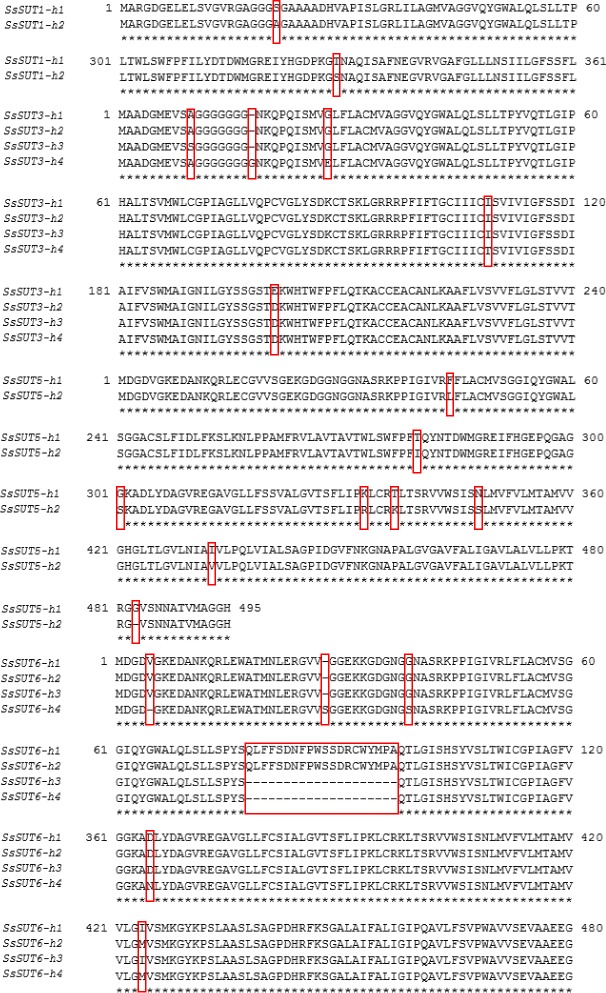
Alignment of the amino acid sequences of *SsSUT* haplotypes. Amino acid sequences of haplotypes were aligned using the DNAMAN program. Similarity in amino acids across all the sequences is indicated by stars. The difference between haplotypes was shown in a red box

Genomic sequence comparisons within the allelic haplotypes from the four *SUTs* revealed that these allelic haplotypes shared very high similarity above 99 %. Slight variations were observed within the allelic haplotypes of *SsSUTs*. The deducted protein sequences were compared within the four *SsSUTs*. The results showed that, for the protein sequence of allelic haplotypes, both *SsSUT3* and *SsSUT5* shared identities of 98.38 %, while, *SsSUT5* and *SsSUT6* had 98.38 and 97.79 % sequence similarity, respectively. The specific amino acids variations were discovered through the alignment of the allelic haplotypes from each *SUT*s. 2, 5, 8 and 5 amino acids within *SsSUT1*, *SsSUT3*, *SsSUT5*, *SsSUT6* respectively were observed to be vary among the allelic haplotypes (Table 4). In addition, in *SsSUT6*, compared with h3 and h4, h1 and h2 had shorter peptides with 20 amino acids deletion caused by the shift of exon structure (Fig. 1). Furthermore, in *SsSUT5*, 7 of the variant amino acids were located at the sixth loop domain (T280I; G301S; K333R and T337K) and transmembrane helixes (F44L; N348S and I433V), indicating that potential functional variation existed among these allelic haplotypes (Fig. 1).

**Table 4:** The variation of deducted amino acid sequences among allelic haplotypes within the four SsSUT

SUT	No. of variations (SsSUT-h1 vs other haplotype)	Amino acid variations
*SsSUT1*	2	S21A,T329S
*SsSUT2*	N/A	-
*SsSUT3*	5	A10S, G18 insertion, G28E, I109T, E200D
*SsSUT4*	N/A	-
*SsSUT5*	8	F44L, T280I, G301S, K333R, T337K, N348S, I433V, G482 insertion
*SsSUT6*	6	V5 deletion, S28 insertion, G39S, Q78…A97 deletion, D345N, I404M

**Fig. 2:**
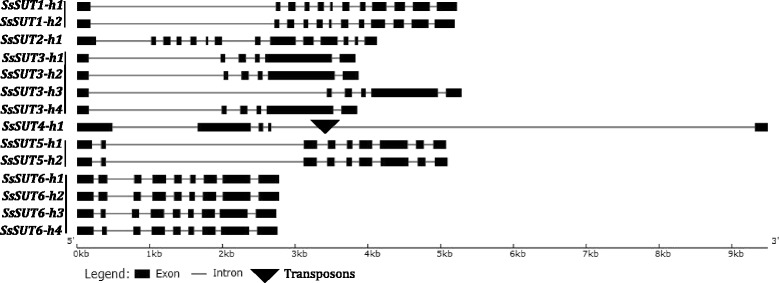
Comparison of the allelic gene structures of *SsSUT*s. Boxes represent exons, triangles represent transposons

In general, gene structures of the *SsSUTs* allelic haplotypes presented high conservation for exon/intron numbers and exon sizes, while, for intron size, in *SsSUT3*, *SsSUT3-h3’s* first intron was larger than other allelic haplotypes; and in *SsSUT6*, both *SsSUT6-h3* and *SsSUT6-h4* contained a smaller second exon than *SsSUT6-h1* and *SsSUT6-h2* (Fig. 2).

**Fig. 3:**
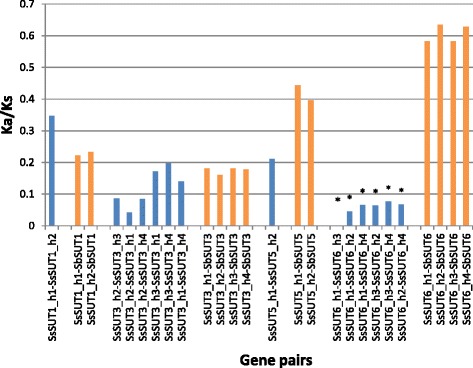
The Ka/Ks of *SsSUT* haplotypes and *SbSUT*-*SsSUT*. The lower value of Ka/Ks was indicated by stars

Nonsynonymous to synonymous substitution ratio (Ka/Ks) was analyzed to investigate evolutionary function constraint in *S.spontaneum*. To identify the evolutionary forces acting on four SUT genes having alleles (*SsSUT1*, *SsSUT3*, *SsSUT5*, *SsSUT6*) (Fig. 3), the Ka/Ks was calculated. Within the coding regions, the Ka/Ks ratio was much less than 1, indicating that purifying selection was the dominant force driving the evolution of *SsSUT* genes.

### Comparative analysis of gene structure between *SsSUT* and other plant *SUT*

**Fig. 4:**
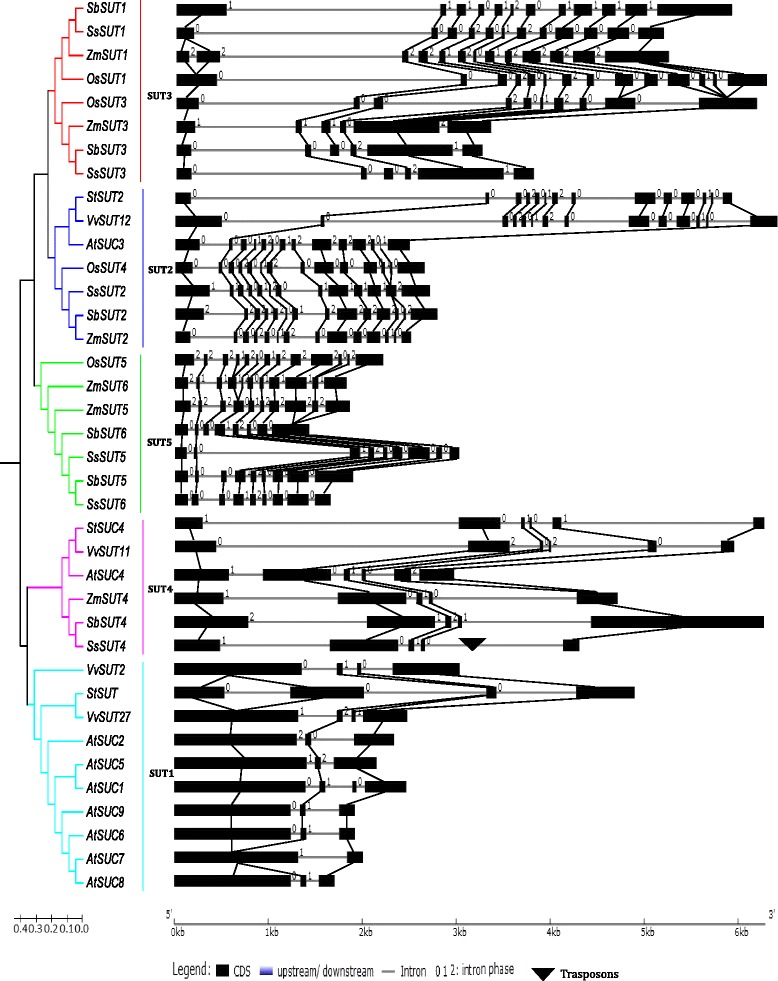
Comparison of the gene structure of the six members of the *SsSUT* gene family. Three monocotyledons (*Zea mays, Sorghum bicolor, Oryza sativa*) and dicotyledons (*Arabidopsis thaliana, Solanum tuberosum, Vitis vinifera*) SUT gene family are also shown for comparison. Boxes represent exons, triangles represent transposons

The gene structures of *SsSUT* family has a great variation with exon numbers ranging from five to fourteen, and their introns were aligned accordance with the GT-AG rule for splicing sites. *SsSUT1, SsSUT3* and *SsSUT4* had larger first introns than the other SUT genes. Both *SsSUT3* and *SsSUT4* only have five exons that were lesser than the other *SsSUTs* (Fig. 4). In addition, comparative analysis of *SsSUT* families suggested that, the fifth exon in *SsSUT3* were presumed to split into 3–4 exons, and the first, second, and fifth exons in *SsSUT4* were presumed to evolve into 2–4 exons (Fig. 4). *SsSUT5* and *SsSUT6* shared highest similarity of exon/intron pattern in spite of the great size variation between the second introns, which correlated to their amino acids similarities (Table 3).

To investigate the evolutionary mechanisms underlying the genesis of gene families, we performed comparative analyses of the *SsSUT* structures with the *SUT* families from sorghum, rice, *Arabidopsis (Arabidopsis thaliana)*, maize, grape (*Vitis vinifera*) and potato (*Solanum tuberosum)* (Fig. 4). The *SUTs* from these species could be divided into five groups SUT1 to SUT5, which were consistent with previous studies [13, 44]. The results showed that *SUTs* in the same group had the similar gene structures. In the dicot specific group SUT1, most of the genes had two to four exons, in which the first exons were large and the second ones were small. In this group, the only exception was *StSUT2,* which had a smaller first exon, due to the possible exon splitting comparing with the other dicot species. In group SUT4 that was closely related to group SUT1, all the genes had first two large exons that were likely originated from SUT1 first exon splitting based on sequence comparison. It was interesting that in SUT4 group, the dicot genes and the monocot genes had siminar number of exons, 5 and 6, respectively, and the sequences of the fifth and sixth exons in dicot were presumed to originated from the monocot fifth exon splitting. Furthermore, based on the genomic and amino acids sequence comparisons among the 16 sequences from the seven plant species in group SUT1 and SUT4, the common ancestral gene of monocots and dicots were suggested to have two exons and the exon members have differentiated in a later period of evolution caused by exon splits and partial exon fusions.

The genes in group containing SUT2 had similar exon number of 13 or 14 for both dicot and monocot species. Gene size expansion due to intron size stretching in dicot plant grape and potato were observed in this group. In the SUT3 group, besides *ZmSUT1* with an additional small intron caused by the first exon splitting, the remaining SUT3 group genes harbor large first introns, which included wheat *TaSUT1D* [12] and tomato LeSUT2 [24]. In contrast to SUT2 group, the SUT3 group had varied number of exons ranging from 6 to 14; among them, *SsSUT3*, *SbSUT3* and *ZmSUT3* had six exons, the other genes in the subfamilies contained 10–14 exons; both of *OsSUT*s in this group had more exons number than their orthologous genes. In monocot specific group SUT5, similar to the high identities shown by the alignment of amino acid sequence of *SsSUT5* and *SsSUT6*, high conservation of exon/intron structures were observed from the schematic representation; *SsSUT5* had larger second exon than the other genes; similar to SUT3 group, *OsSUT* had more exon number than their orthologous genes. Scrutiny of the exon/intron structure of the 22 genes in branch with SUT3/SUT2/SUT5 revealed that the exons could be corresponding to six exons, in which the first and the last two exons were observed to be fused/spited. These results suggested that the genes in this branch might originate from common ancestral gene containing six exons for both monocots and dicots.

### Phylogenetic analysis of *SsSUT* and other plant *SUT* homologs

**Fig. 5:**
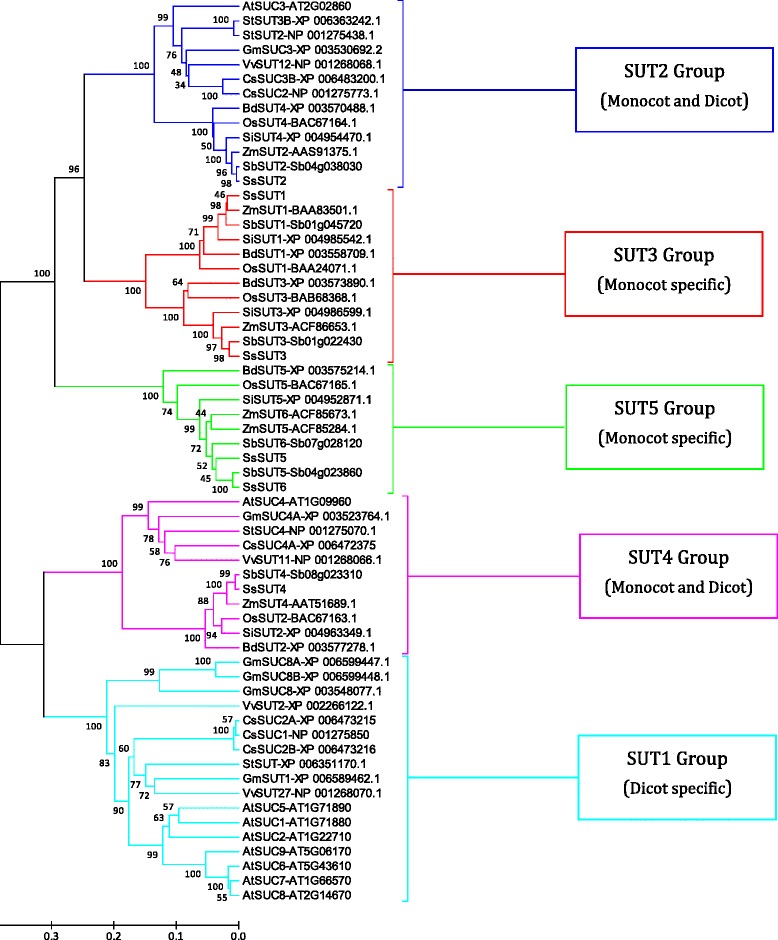
Phylogenetic analysis of *SsSUT* and other plant *SUT* homologs. Unrooted phylogenetic tree of plant SUT proteins constructed using the neighbour-joining method with MEGA 5.2.1 program. *ZmSUT*: *Zea mays*, *SbSUT*: *Sorghum bicolor*, *BdSUT*: *Brachypodium distachyon*, *OsSUT*: *Oryza sativa*, *AtSUT*: *Arabidopsis thaliana*, *CsSUT*: *Citrus sinensis*, *GmSUT*: *Glycine max*, *StSUT*: *Solanum tuberosum*,*VvSUT*: *Vitis vinifera, SiSUT: Setaria italica*, *SsSUT*:*Saccharum spontaneum*

To comprehensively analyze the evolutionary relationships of *SUT* families between *S. spontaneum* and other plant species, we aligned 62 plant amino acid sequences from 5 dicots and 6 monocots including *S.spontaneum,* using ClustalX to construct an unrooted tree with Neighbor-Joining method (Fig. 5). Same as the distribution above, the *SUTs* were phylogenetically distributed into five groups, SUT1, SUT2, SUT3, SUT4 and SUT5, respectively. Among these five groups, SUT1 genes are only found in dicotyledonous, in contract, genes in SUT3 and SUT5 groups are only from monocotyledonous. Whereas, the remaining two groups, SUT2 and SUT4, are found in both dicot and monocot, and could be well classified into two distinct subclades. These results strongly suggested that plant SUTs were diverged from a recent evolutionary event after the common ancestor of dicots and monocots. In addition, the SUT families could be divided into two branches in the phylogenetic tree, with SUT1 and SUT4 groups in one branch and the other three groups in another branch, indicting two ancestral genes were the origins of SUTs in both dicot and monocot.

**Fig. 6:**
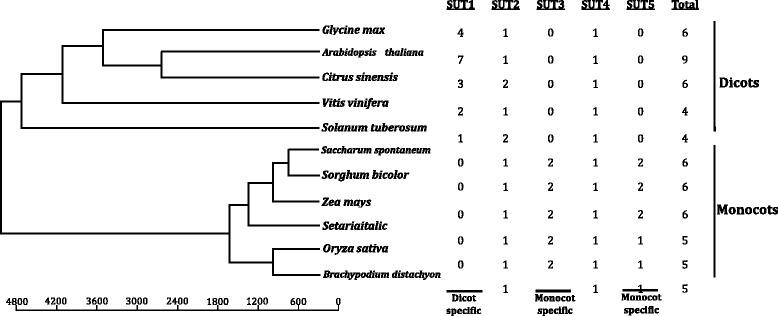
The distribution of *SUT* family member in monocotyledon (6 species) and dicotyledon (5 species)

In dicot specific SUT1 group, the paralogous genes from each of the dicot species were observed to be closely related, indicting recent gene duplications after the divergence of dicotyledons (Fig. 5). In addition, both *SUT* gene number and sequences had great variations among the dicot plant species, suggesting rapid evolutionary dynamics exist in the dicotyledonous SUT families (Figs. 5 and 6). In SUT4 group, all dicots and monocots had only one gene member, which the phylogenetic distribution were generally consistent with the plant species taxonomy (Figs. 5 and 6). In SUT2, as description above, the genes from dicot and monocot species could be further classified into two subclades, respectively. In this group, dicot plants have d 1-2 gene members, while, all the monocot plants only hadone gene member, which suggestted that dicot plants SUT2 group were undergoing expansion. In the monocot specific SUT3, the examined monocot species consisted of two paralogous genes from two separated clades, suggesting the gene duplication event occurred before the divergence of these dicot plants. In contract to SUT3 group, in SUT5 group, recent gene duplication events were observed in the *Andropogoneae* plants (*S. sponteneum*, *Sorghum bicolor* and *Zea Mays*) and *Bambusa oldhamiias* shown by the closest phylogenetic distribution of genes within these plant species.

In the *SsSUT* family, *SsSUT4* was the solo member in a branch, but *SsSUT1, SsSUT2*, *SsSUT3*, *SsSUT5* and *SsSUT6* were clustered together and shared a more recent common ancestral gene. Thus, *SsSUT4* was suggested to be the oldest gene, while, both of *SsSUT5* and *SsSUT6* should be younger than the splitting of *Trib*, *Andropogoneae* Dumort and *Zea Mays*. In addition, *SsSUT5* and *SsSUT6* were observed to be undergoing rapid evolution as shown by lower amino acids sequences similarity than other orthologous genes between the sorghum and *Saccharum* (Table 2).

### Gene expression of *SUTs* among three *Saccharum* species

**Fig. 7:**
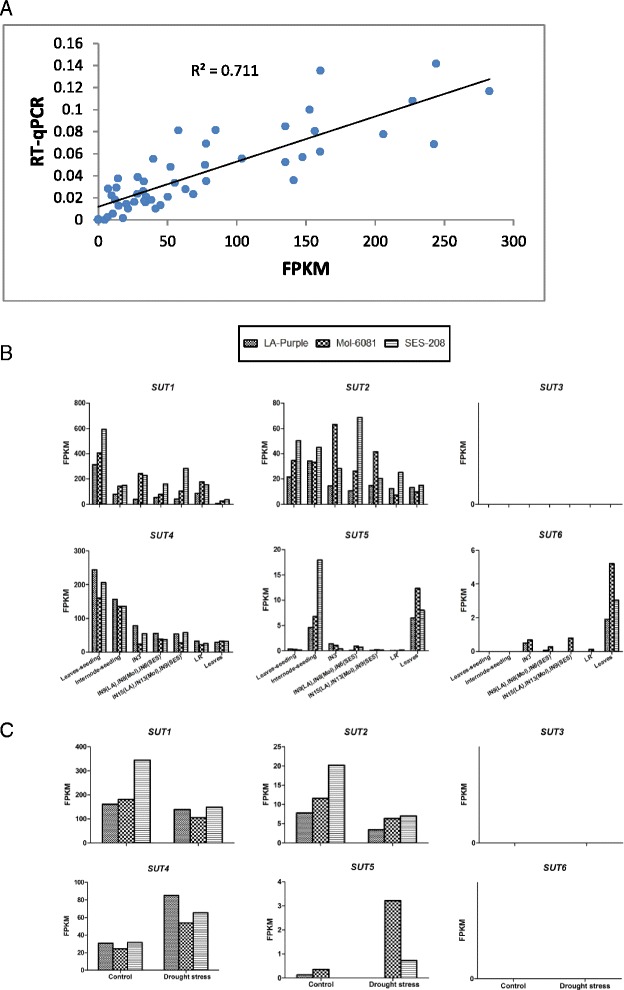
The SUT gene family expression based on RPKM in different tissues of different stage in three *Saccharum* species. The expression level of 6 SUT genes in seeding stage and mature stage of nature condition. **a** correlation coefficient between RNAseq (X-axe) and qRT-PCR (Y-axe) of six SUT genes. **b**, and in seeding stage of drought stress condition (**c**). IN, internode; LR, leaf roll. Internnodes 3, 9, 15, internodes 3, 8, 13 and internodes 3, 6, 9 were from *Saccharum officinarum* (LA-Purple), *Saccharum robustum* (Molokai6081) and *Saccharum spontaneum* (SES208), respectively

To investigate the possible physiological functions for SUTs, we performed comparative transcriptome profiling among three *Saccharum* species at different developmental stages of seedling and five different tissues from the mature leaf (mature and leaf roll) and stalks (mature, maturing and immature) by using RNA-seq method. The RNA-seq results were verified by qRT-PCR in three tissues (leaf roll, mature stalk and maturing stalk) from each of the three Saccharum speices (Additional file 4 and Additional file 5). There is a significant positive relationship ( R2 = 0.711 and *p* < 0.001) between the Reads Per Kilobases per Million reads (RPKM) based on RNA-seq and the relative expression level based on qRT-PCR (Fig. 7a).

In SUT families, the transcription of *SUT3* was undetectable in all the examined tissues from *Sacchaurm* plants, which was consistent with that of *SUT3* in sorghum [13]. The other gene expression levels had significant variations with a clear trend of transcript levels from higher to lower *SUT1*, *SUT4*, *SUT2*, *SUT5* and *SUT6*. Of them, *SUT1* and *SUT4* had predominant expression levels among the gene families, indicating that the two genes were the fundamental members in SUT families.

*SUT1* transcripts were abundant in both source and sink organs, and were the most expressed gene in all tissues except for the mature leaf, indicating *SUT1* was the key member. The gene displayed a higher expression level in sink tissues (stem) than the source tissue (leaf) in mature plant. However, it was the opposite in seedling plants, suggesting the gene was more important in phloem loading for seedlings than the mature plant. Comparing with both *S. sponteanum* and *S. robustum*, *SUT1* displayed a lower expression level in the high sucrose *S. officinarum* (LA-Purple), in all examined tissues. In addition, under drought stress, *SUT1* was observed to be down regulated in *S. robustum*.

*SUT2* was expressed in all the organs examined for the three *Saccharum* species. In the seedling plants, similar expressions were observed in both stem and leaves among these *Saccharum* species. In the mature plants, *SUT2* displayed a lower expression level in the source tissue of leave and leafroll for two lower sucrose accumulating *Saccahurm* species, *S. sponteneum* and *S. robustum*, while, high sugar *S. officinarum* had a more uniform *SUT2* expression level in all of the examined tissues (Fig. 7b). Under drought stress, twofold lower levels of expressions were observed in the three *Saccharum* species than their control (Fig. 7c).

*SUT4* had higher expression level in the seedling than the mature plants (Fig. 7b), suggesting it might contribute more for sucrose loading at the early age of plant development; whereas, *SUT4* displayed similar expression level in the examined tissues from both seedling and mature plants. Obviously, *SUT4* was not correlated to the sucrose content differential among the *Saccharum* species. Under drought stress, in contrast to *SUT1* and *SUT2*, *SUT4* was up regulated in the leaf of three *Saccharum* species.

*SUT5* displayed dramatically higher expression level in sink tissues (stem) than in source (leave) in the seeding plant; in contract, in the mature plants, the expression level were lower in leave than the other tissues. Similar to *SUT4*, *SUT5* showed up-regulation under water stress. *SUT6* was undetectable in the seeding plants and had similar expression pattern as *SUT5* in the mature plants that gene expression exhibited higher in leave than the other tissues.

## Discussions

Genomic study for the gene families is the first step toward the gene functional study. However, the identifications of gene family in sugarcane is still a formidable challenge caused by its complex genomes. Recently, the whole genome sequencing of sorghum and other relative species of *Saccharum* provides the references for comparative genomics to identify the gene families in *Saccharum* species. In previous studies, based on comparative genomics, the gene families of phosphoenol pyruvate carboxylase gene [45], sucrose synthase [34], sucrose phosphate synthase [46], ATP-dependent phosphofructokinase [37] were identified by using the EST database, DNA fragment, and cDNA cloning. However, none of these studies has investigated on the genomics of whole gene families because of the lack of genomic sequences for sugarcane. Our study through comparative genomics and BACs sequencing is the first report for the structure of a gene family and their gene allelic haplotypes in *Saccharum*.

### Evolutionary conservation and divergence of *SsSUT*

Plant SUTs had been well documented in previous studies for gene phylogenetic analyses two classifications [11, 13, 44, 47, 48]. One classification was to divide plant SUTs into three types, type I, type II and type III, with the reference of *Arabidopsis* [11, 48], in which, type I and type II SUTs were localized to the plasma membrane, while type III SUTs were associated with vacuolar membrane [48]. Another classification was to group the SUTs into five groups SUT1, SUT2, SUT3, SUT4 and SUT5 [13, 44, 49]. Comparing these two classifications, SUT1 was included in type I, SUT2 was in type II, and SUT3, SUT4 and SUT5 were in type III. The former classification is likely associated with dicot plants studies, while, the latter is used for both dicot and monocot plants, especially for gene evolution studies. In this study, we used large amount of plant *SUT*s for phylogenetic analysis, and the results confirmed the later classification, by revealing the existence of one dicot specific and two monocots specific groups and the independence evolution process in plant SUT families (Fig. 5). Furthermore, the comparative analysis of these plant *SUT*s showed that genes in SUT3 group were more conservative than the genes in SUT5 group, and further provided the direct evidence of the recent duplication event occurred after monocot/dicot divergence. The evolution history of *SsSUTs*, which was sorted by age in duplicated descending order, *SsSUT4, SsSUT2*, *SsSUT2*/*SsSUT3*, and *SsSUT5/SsSUT6*.

The comparative analyses could be used to predict the number of SUT gene family members in *Saccharum*. Without the whole genome sequences for *Saccharum*, it could be debatable to conclude that we have discovered all *SUTs* in *Sacchaurm,* in spite of our high coverage BACs libraries. In this study, both phylogenetic analysis and sequences comparison revealed that *Saccharum spontaneum* and *Sorghum bicolor* were both composed of six *SUTs* members and each gene pairs were orthologous between the two species (Figs. 5 and 6). Beside SUT1 group, which was dicot specific, monocot plants have the same gene numbers in SUT2, SUT3 and SUT4 groups, the remaining group SUT5 had the same gene number in *Andropogoneae* plants, which were found to be the result of a recent duplication (Fig. 6). Therefore, it is less likely that any SUT gene duplication events had occurred after the diverging of sorghum and *Saccharum,* knowing no additional gene in this study. Hence, we concluded that the six *SsSUTs* comprise the *SUT* family in the *Saccarhum spontanum* genome. Further experiments such as Southern Blot could be used to verify the conclusion.

The exon–intron structure differences were demonstrated to be accomplished by three main types of mechanisms, exon/intron gain/loss, exonization/pseudoexonization, and insertion/deletion [50]. In this study, comparative analyses of gene structures for *SsSUT* made it possible to evaluate the *SUT* gene structure evolution in plant (Fig. 4). All SUTs genes, including *SsSUT*s, had a great variation of exon numbers, ranging from 2 to 14. Comparing with the gene structure variation, the protein sequences were more conserved among the paralogous genes in *Sacchaurm*, as shown by all SUT members containing 12 membrane-spanning helices and similar protein sizes. A common feature of plant SUTs was that the 12 membrane-spanning helices were distributed roughly uniformly in the deducted peptides of each gene in conserved position. Thus, the gene structure evolution after these plant divergences did not cause significant coding region variations. Therefore, the *SUT* structures variations were mainly evolved from intron gain/lost and insertion/deletion but not from exonization/pseudoexonization.

*Sorghum* is one of the closest relative diploid genera of *Sacchaurm*. Comparative analysis of the orthologous between *SsSUTs* and *SbSUTs* made it possible to investigate the specific evolutionary events after the polyploidzation of *Saccharum*. In *SsSUTs*, *SsSUT5* harbored a much larger second intron than its closest paralogous *SsSUT6* and the other orthologs (Fig. 4). Similarly, *SsSUT3* contained a larger first intron than its orthologous gene *SbSUT2*. In addition, *SsSUT4* were observed to have a putative TE (Transposable elements) insertion in the last intron (Fig. 4). Our results suggested that the *SsSUT* families were undergoing gene extension following polyploidization in *S.spontaneum*. The estimated monoploid genome size of *S. spontaneum* (843 Mb) and *S. Officinarum* (985 Mb) were both larger than the monoploid genome size of sorghum at 760 Mb [34], supporting the conclusion that the genome of *Saccharum* expanded in general after polyploidization. Our results demonstrated the first case that expansion in intron regions of a gene families contribute to genome expansion in *Sacchaurm*.

In this study, allelic haplotype sequences for *SsSUT1*, *3*, *5* and *6* were comparatively analyzed for gene structures and Ka/Ks. Relative conservative gene structures were observed among allelic haplotypes within each of the four *SsSUTs*, whereas, *SsSUT3-h3* contained a larger first intron than the other three haplotypes, both *SsSUT6-h1* and *SsSUT6-h2* had larger second exons (Fig. 2). In addition, the Ka/Ks ratios, which are all under 0.4, revealed that all the SUTs allelic haplotypes were under strong purifying selection (Fig. 3). Multiple alleles in polypoidy are considered to be functional redundant at the time of origin [51–53]; the conservation and constraint purification within the allelic haplotypes of *SsSUTs* were likely due to the key function of SUT in *Saccharum*. It would be worthy to note that the transcription of *SsSUT3* was undetectable in the examined tissues while its haplotypes were under constrain selection, indicating that *SsSUT3* might have a necessary function for *Sacchaurm*. In paleopolyploids [54–57], and recent allopolyploid species, such as wheat [58, 59] and Tragopogon [60, 61], eliminations and pseudogenizations of key functional genes after polyploidzation have been well documented. The allelic gene variations supposed to the key topics for studying the genome dosage of *Sacchaurm* species and further investigation for them would provide the foundation to understand the molecular basis of sugarcane genetics.

### Gene expression and functions of *SUTs* in *Saccharum*

Examination of SUT gene expressions in *Saccharum* species in source and sink tissues of seedling and mature plants proves an insightful indication regarding the roles of gene functions. Previous studies of gene expression were mostly done on *Saccharum* hybrids with combined genetic background of *S.officinarum* and *S.sponteneum*. To simplify genetic backgrounds, in this study, three *Saccharum* species, high-sucrose *S.offcinarum*, low-sucrose *S.robustum* (the potential domestic progenitor of *S.officinarum*) and stress-tolerant *S.spontaneum* were used for studying the *SUTs* expression profiles.

*SUT1* was the only gene in SUT family, which had been well documented in *Saccharum* hybrid [30, 31, 33, 62]. In these previous studies, *ShSUT1* had been shown to have high expression level in premature stem tissue, and decreased expression level in mature internodes [33]. *ShSUT1* was demonstrated to be highly selective for sucrose, inhibited by sucralose and had the function in loading sucrose from the vascular tissue into the stem parenchyma cells [30, 33]. Consistent with previous study [30], our results suggested that the expression of *SUT1* was higher in mature stems than the mature leave from all three *Saccharum* species (Fig. 7b), and thus supported the previous conclusions. Moreover, *Saccharum SUT1s* displayed much higher expression level in the seedling leave tissues than in both of the seedling stems and all the mature tissues, suggesting *SUT1* may play an enhanced role in directing sucrose in source tissue before sucrose accumulation in *Sacchaurm* species. Similar to the expression of *SbSUT1* in sorghum [13], the *SUT* family expression analysis revealed that *SUT1* was the most expressed among SUTs in *Saccharum* species. Nevertheless, the only direct evidence for the SUT1 function was from the *Saccharum* close related maize *sut1* mutant, which exhibited a phenotype of shorter stature and carbohydrate accumulation in their source leaves [63]. *SUT1* in *Saccharum* may have similar function as *ZmSUT1* since the two orthologs shared high sequence similarity.

Comparative analyses of *SUT* expression among the three *Saccharum* species revealed that *SUT1* had lower expression level in all tissue types from high sugar species (*S.officinarum*) than the lower sugar species (*S.spontaneum* and *S.robustum*), which was consistent to sorghum that *SUT1* had lower gene expression in high sucrose *Rio.* than in the grain type genotype *RTx623* [13]. Similar to *SUT1*, besides the leaf tissues, *SUT2* was more abundant in *S.spontaneum* and *S.robustum* than in *S.officinarum* (Fig. 7b). These results supported the notion that sinks demand in the mature plant might be stronger than in the lower sucrose content plants of *Andropogoneae* tribe.

**Fig. 8:**
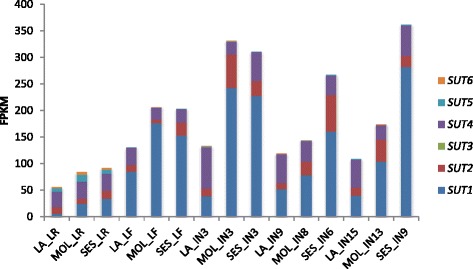
Total accumulative expression level of the *SUTs* in tissues and in stage of three *Saccharum* species. IN, internode; LR, leaf roll. Internnodes 3, 9, 15, internodes 3, 8, 13 and internodes 3, 6, 9 were from Saccharum officinarum LA-Purple, Saccharum robustum Molokai6081 and Saccharum spontaneum SES208 respectively

We compared the expression level of *SUTs* of all *Saccharum* species in both mature and seedling plant. In mature plants, *SUT1* had higher expression level in sink than source tissues; in contrast, *SUT1* had a lower expression level in sink than source tissues. *SUT4* showed a higher expression level in seedling than the mature plants. *SUT1* and *SUT4* accounted for above 70 % of transcripts in this gene family (Fig. 8). These results indicated that sucrose transport were active before the sucrose accumulation and both *SUT1* and *SUT4* were involved in the plant development in *Saccharum*.

In the *Saccharum* species, *SUT4* showed similar expression level in both seedling and mature stage tissues. These results were different from its close orthologs including *OsSUT2* [64], *PtaSUT4* [8], and *SbSUT2* [13]. Thus, SUT4 might contribute to the characteristics of sucrose accumulation in *Saccharum* species. The orthologous of *SUT4* phylogenetic group was proved to be localized in the tonoplast [64–66]. In model plant *Oryza sativa* [11] and *Arabidopsis* [67], SUT4 played a role for transporting sucrose from mesophyll vacuoles to their cytoplasm. This information may not be sufficient for discovering the gene function of *SUT4,* but is an indication for further functional study of *SUT4* in sugarcane.

Both *SUT5* and *SUT6* in SUT5 group (Fig. 5) were revealed to be recent duplication and through rapid evolution accompanied by the multiple amino acid differences in their allelic haplotypes (Table 4), suggesting that these two genes may have similar gene expression profiles. Gene expression analysis showed that these two genes have much lower gene expression level among the SUTs families and had similar gene expression profiles in the mature tissues (Fig. 7b), supporting that the two genes were derived from a recent duplication. Nevertheless, in the seedling plants, *SUT6* was absent while *SUT5* had higher expression in stems than leaves (Fig. 7b), hence, SUT5 may contribute to phloem loading before the sucrose accumulation in *Saccharum.* In *sorghum*, great variation of gene expression level for *SbSUT5* and *SbSUT6* were observed among the tissues from of vegetative stages and anthesis [13]. Of them, *SbSUT5* showed higher expression in spikelet tissue and inflorescence, and thus was suggested to play a role for inflorescence development; similarly, *SUT6* in *Saccharum* species was more abundant in leaves than the other tissues [13]. Based on above genomic analysis, *SUT5* and *SUT6* have gone through rapid evolution after the split of *Sorghum* and *Saccharum*, suggesting that these two genes have functional divergence between these two species. These two gene expression profiles in *Saccharum* and *Sorghum* were different from their closest orthologous *OsSUT5*, which exhibited broad expression level across source and sink tissues as well as in filling rice grains [11]. Phylogenetic and comparative analysis revealed that there was a single *SUT* in group SUT5 from rice. These differences can be explained by the single gene *OsSUT5* in rice response for function of two duplication genes in the *Andropogoneae* tribe.

Soluble sugar such as sucrose usually increases in plant under drought stress. to identify which SUT gene responsible to drought stress, we examined the *SUTs* expression level under drought stress in the seedling plant leaves of three *Saccharum* species. Under drought stress, in the four detectable *SUTs* in the seeding, *SUT1* and *SUT2* were down regulated, in contrast, *SUT4* and *SUT5* were up regulated, indicating that the SUT4 and SUT5 are important in response to drought stress and may involved in transporting sugar into cell for osmotic adjustment. SUT families in *Saccharum* presented a great gene expression diversity in response to drought stress. *SUT4* was the predominant expression member in the SUTs families in *Saccharum*, therefore, the up-regulation of *SUT4* expression resulted in the higher total *SUTs* transcript level. A possible explanation for this phenomenon could be that the source tissue reduced the sucrose product level under drought stress thus down regulated the *SUT* expression level. An expression profile for *Saccharum* plants under water stress with different time points could be further used for verifying this notion. Similar experiment was performed for the five SUT members in rice, which revealed that *OsSUT2* was only member display up-regulated during drought and salinity treatments [68]. Therefore, it is most likely that plant SUT gene members possess diversity pattern in response to stress tolerance.

## Conclusion

In this study, we presented the first report of a gene family consists of six *SUTs* in *Saccharum*. We provided the comprehensive evaluation of the evolutionary genesis, gene allelic haplotypes, phylogenetic relationships, gene structure, and gene expression pattern for the SUT gene family in *Saccharum* species. Our results revealed that *SsSUT*5 and *SsSUT6* were recent duplication genes companied by rapid evolution, while, *SsSUT2* and *SsSUT*4 were the ancient members in the families. Gene size extensions caused by sequence insertions in introns were observed in the SUT families. Despite the high polyploidy level, the examined SUTs exhibited conserved gene structures and amino acid sequences among the allelic haplotypes. Both *SUT1* and *SUT4* had predominant expression in *Saccharum* SUT families. *SUT1,* which displayed lower expression in the high sucrose species *S.officinarum,* might be involved in saccharide unloading in the sink tissue of mature plant, phloem loading in early developmental stage of *Saccharum. SUT2* likely contributed to both phloem loading and sink development. *SUT4* was more important at early developmental stage than in mature plants. Both *SUT5* and *SUT6* had lower expression level than other gene members, and had higher expression in source leaves than in other tissues, thus, supposed to play roles in phloem loading. In the seedling plant leaves with drought stress treatment, four genes *SUT1*, *SUT2*, *SUT4* and *SUT5* were detectable, among which, *SUT1* and *SUT4* were down regulated, while, *SUT2* and *SUT5* were up regulated. To further reveal these genes’ roles under stress, experiments such as, characterizing the spatio-temporal expression dissection, enzyme activity assay, and gene editing technology like CRISPR-Cas9 system, would be necessary. The results offered useful foundation and framework for future research for understanding the physiological roles for each SUT gene and molecular mechanisms of sucrose metabolism in sugarcane.

## Availability of supporting data

The 14 sequences of *SsSUTs*(allele haplotypes) were deposited into Genbank (accession numbers: KT284760-KT284773).

Phylogenetic data (alignments and phylogenetic trees) have been deposited to TreeBase and are accessible via the URL: http://purl.org/phylo/treebase/phylows/study/TB2:S18751.

## Additional files

Additional file 1:
**The probe primers for SUT BAC hybridization in** ***S. spontaneum*** **.** (DOC 32 kb) Additional file 2:
**BLAST results for** ***SsSUTs*** **EST in NCBI database.** (DOC 32 kb) Additional file 3:
**Alignment of the amino acid sequences of** ***SsSUT*** **haplotypes.** (DOC 114 kb) Additional file 4:
**The qRT-PCR primers for** ***SUTs*** **in this study.** (DOC 32 kb) Additional file 5:
**qRT-PCR verification of SUT gene expressions in partial issues of three Saccharum species.** (DOC 824 kb)

## Abbreviations

BACs: bacterial artificial chromosomes
eEF-1a: Eukaryotic elongation factor 1a
EST: Expressed Sequence Tag
GAPDH: glyceraldehyde-3-phosphate dehydrogenase gene
RPKM: Reads Per Kilobasesper Million reads
SUTs: Sucrose transporters
