# Supplementary material for: Structure, phylogeny, allelic haplotypes and expression of sucrose transporter gene families in Saccharum
Source: BMC Genomics. 2016 Feb 1;17:88. doi: 10.1186/s12864-016-2419-6 (PMC4736615; doi:10.1186/s12864-016-2419-6)
Supplement: Additional file 1: — The probe primers for SUT BAC hybridization in S. spontaneum . (DOC 32 kb) [file 12864_2016_2419_MOESM1_ESM.doc]

***Additional file 1. The primers for SUT BAC hybridization probes in S.spontaneum***

| **Gene name** | **Forward** | **Reverse** | **Amplicons** |
| --- | --- | --- | --- |
| *SsSUT1* | CAGTGATAGGTGTACAGCAAGATG | GGAGAAGTCAAGGAGCCAAAAC | 452 bp |
| *SsSUT2* | CTGAAGAGATCCCACTAGAGCC | CCATCCAGTCAGTGTCAAAAAG | 460 bp |
| *SsSUT3* | CCTCCGACATTGGTTACGC | TTGGTCTGCAGGAACGGG | 404 bp |
| *SsSUT4* | TGCTCAACTCTGTCGTCCTT | GACCAAGATTTTCAACACGACT | 398 bp |
| *SsSUT5* | CAGGTGGCTTCCTTGGCTCA | GGGGGAAGGTTCTTGAGGCT | 343 bp |
| *SsSUT6* | CAGCAACTCGACAAGGACAAC | GGAGGAAGGAGGTGACGC | 383 bp |
