# Supplementary material for: Structure, phylogeny, allelic haplotypes and expression of sucrose transporter gene families in Saccharum
Source: BMC Genomics. 2016 Feb 1;17:88. doi: 10.1186/s12864-016-2419-6 (PMC4736615; doi:10.1186/s12864-016-2419-6)
Supplement: Additional file 2: — BLAST results for SsSUTs EST in NCBI database. (DOC 32 kb) [file 12864_2016_2419_MOESM2_ESM.doc]

**Additional file 2. BLAST results for *SsSUTs* EST in NCBI database**

| **Gene name** | **The number of aligned EST** | **The Accession of aligned EST** |
| --- | --- | --- |
| ***SsSUT1*** | 7 | **CA195914.1 DN194145.1 BU925792.1 BU925783.1 CA220149.1 CA163799.1**  **CA222528.1** |
| ***SsSUT2*** | 8 | **CA207180.1 CA074770.1 CA083475.1 CA261327.1 CA198558.1 CA295276.1**  **CA295342.1 CA079114.1** |
| ***SsSUT3*** | 0 |  |
| ***SsSUT4*** | 10 | **CA149288.1 CA194347.1 CA182692.1 CA120749.1 CA181808.1 CA183875.1**  **CA249286.1 CA222438.1 CA208257.1 CA232776.1** |
| ***SsSUT5*** | 1 | **CA292306.1** |
| ***SsSUT6*** | 2 | **CA210755.1 CA174528.1** |
