# Supplementary material for: Structure, phylogeny, allelic haplotypes and expression of sucrose transporter gene families in Saccharum
Source: BMC Genomics. 2016 Feb 1;17:88. doi: 10.1186/s12864-016-2419-6 (PMC4736615; doi:10.1186/s12864-016-2419-6)

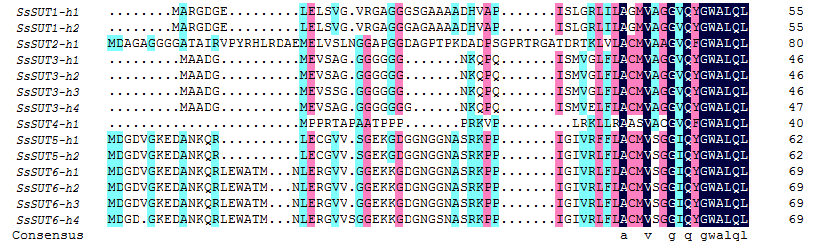

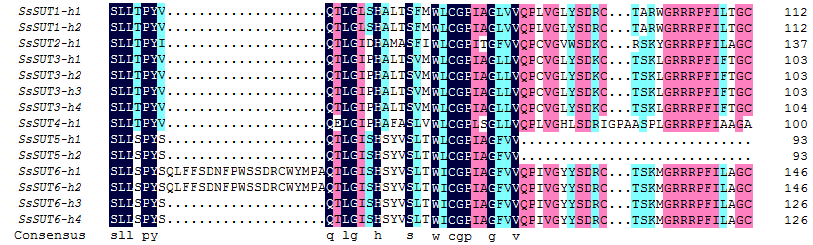

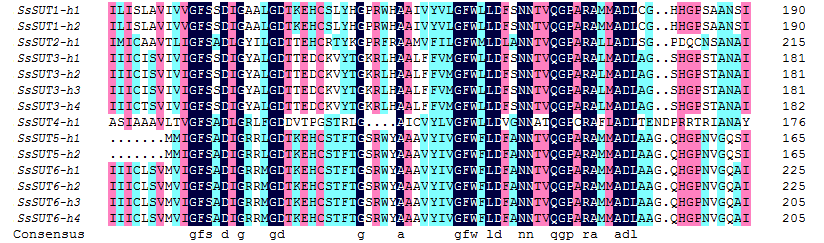

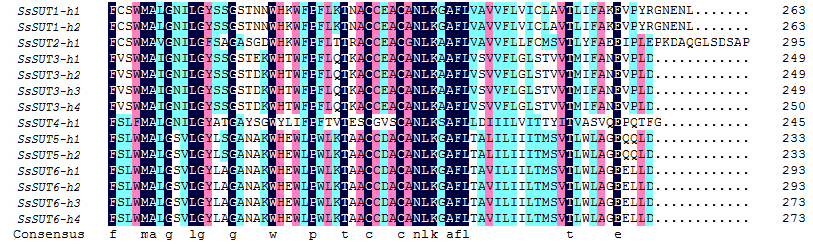


**Additional file 3. Alignment of the amino acid sequences of *SsSUT* haplotypes.** Amino acid sequences of haplotypes were aligned using the DNAMAN program. Similarity in amino acids across all the sequences is indicated by lowercase. The MFS domain, coined as common features of all members of the MFS superfamily to which all the known plant SUTs belong, are underlined.


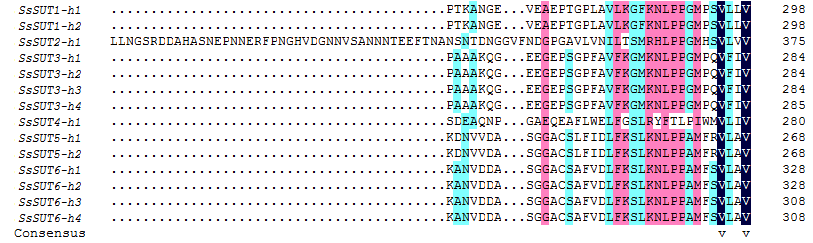

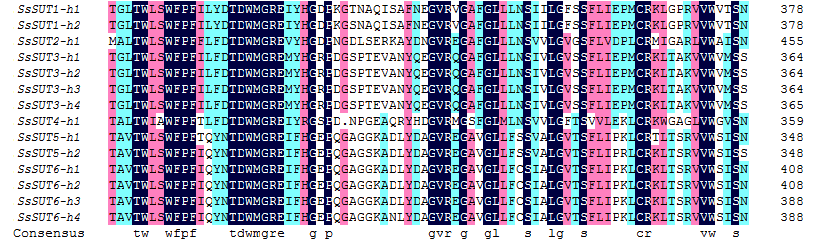

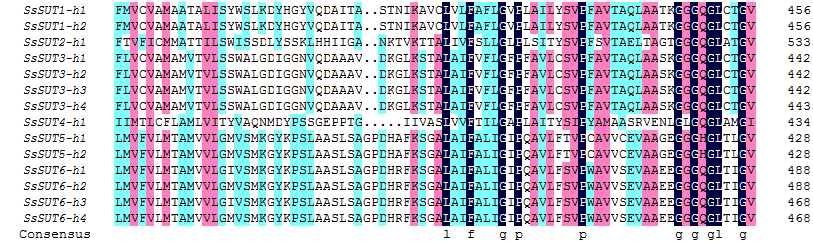

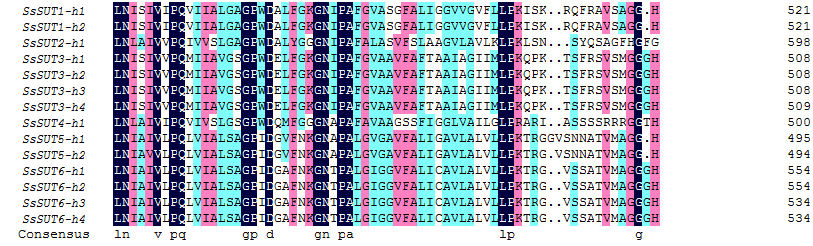

Supplement: Additional file 3: — Alignment of the amino acid sequences of SsSUT haplotypes. (DOC 114 kb) [file 12864_2016_2419_MOESM3_ESM.doc]
