# Supplementary material for: Structure, phylogeny, allelic haplotypes and expression of sucrose transporter gene families in Saccharum
Source: BMC Genomics. 2016 Feb 1;17:88. doi: 10.1186/s12864-016-2419-6 (PMC4736615; doi:10.1186/s12864-016-2419-6)
Supplement: Additional file 4: — The qRT-PCR primers for SUTs in this study. (DOC 32 kb) [file 12864_2016_2419_MOESM4_ESM.doc]

**Additional file 4.** The primers for qRT-PCR verification of *SUT* in three *Saccharum* species

| **Gene name** | **Forward** | **Reverse** |
| --- | --- | --- |
| *GAPDH* | CACGGCCACTGGAAGCA | TCCTCAGGGTTCCTGATGCC |
| *eEF-1a* | TTTCACACTTGGAGTGAAGCAGAT | GACTTCCTTCACAATCTCATCATAA |
| *SUT1* | GGTCCTATTGCTGGCTTAGTG | GACAGCAAGGCTGATGAGAATA |
| *SUT2* | TGGACCTGGCAAACAATACA | CAGCCATCCATGAGCAGAATA |
| *SUT3* | ACGGAGGCCATTCATCTTTAC | GTAACCAATGTCGGAGGAGAAG |
| *SUT4* | GGCTGCTAGTCGTGTTGAAA | CCACTACCAAGCGACACAATAA |
| *SUT5* | GCATCGTCCGGTTCTTCTT | ATCCCAAGAGTCTGAGAGTAGG |
| *SUT6* | CATGGTCGTCTGATCGATGTT | ACCACAAATCCGGCGATAG |
