# Supplementary material for: Structure, phylogeny, allelic haplotypes and expression of sucrose transporter gene families in Saccharum
Source: BMC Genomics. 2016 Feb 1;17:88. doi: 10.1186/s12864-016-2419-6 (PMC4736615; doi:10.1186/s12864-016-2419-6)

**Additional file 5. qRT-PCR verification of six SUT genes in partial tissues of three *Saccharum* species.** IN, internode; LR,leaf roll. Internnodes 3, 9, 15,internodes 3, 8, 13 and internodes 3, 6, 9 were from *Saccharum officinarum* LA-Purple, *Saccharum robustum* Molokai6081 and *Saccharum spontaneum* SES208 respectively.


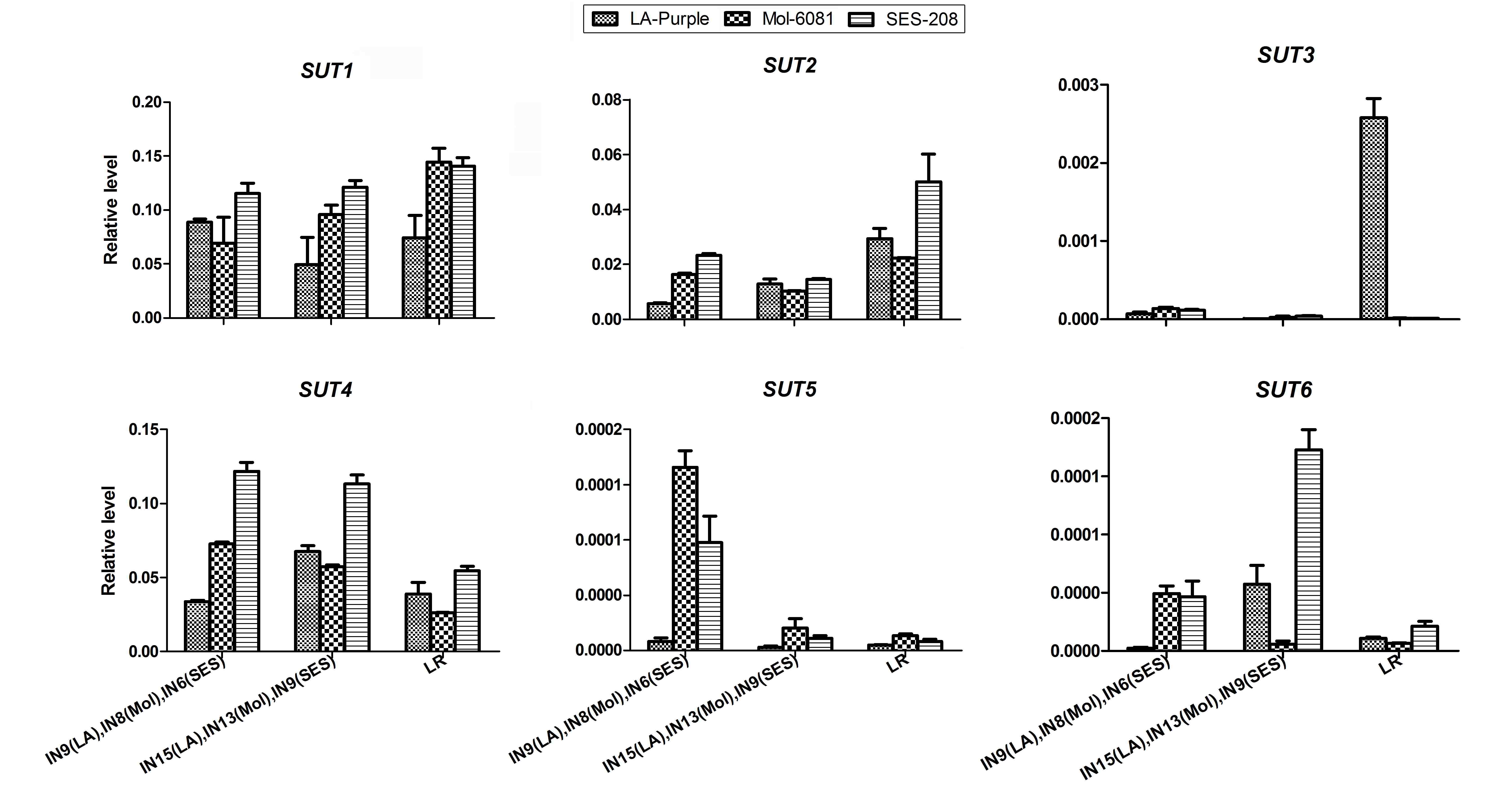

Supplement: Additional file 5: — qRT-PCR verification of SUT gene expressions in partial issues of three Saccharum species. (DOC 824 kb) [file 12864_2016_2419_MOESM5_ESM.doc]
